# Sigma-1 Receptor is a Pharmacological Target to Promote Neuroprotection in the SOD1^G93A^ ALS Mice

**DOI:** 10.3389/fphar.2021.780588

**Published:** 2021-12-10

**Authors:** Núria Gaja-Capdevila, Neus Hernández, Xavier Navarro, Mireia Herrando-Grabulosa

**Affiliations:** ^1^ Institute of Neurosciences, Department Cell Biology, Physiology and Immunology, Universitat Autònoma de Barcelona, Bellaterra, Spain; ^2^ Centro de Investigación Biomédica en Red Sobre Enfermedades Neurodegenerativas (CIBERNED), Madrid, Spain

**Keywords:** sigma-1 receptor, amyotrophic lateral sclerosis, motoneuron, SOD1 G93A transgenic, neurodegenerative disease

## Abstract

Amyotrophic Lateral Sclerosis (ALS) is a neurodegenerative disorder characterized by the death of motoneurons (MNs) with a poor prognosis. There is no available cure, thus, novel therapeutic targets are urgently needed. Sigma-1 receptor (Sig-1R) has been reported as a target to treat experimental models of degenerative diseases and, importantly, mutations in the Sig-1R gene cause several types of motoneuron disease (MND). In this study we compared the potential therapeutic effect of three Sig-1R ligands, the agonists PRE-084 and SA4503 and the antagonist BD1063, in the SOD1^G93A^ mouse model of ALS. Pharmacological administration was from 8 to 16 weeks of age, and the neuromuscular function and disease progression were evaluated using nerve conduction and rotarod tests. At the end of follow up (16 weeks), samples were harvested for histological and molecular analyses. The results showed that PRE-084, as well as BD1063 treatment was able to preserve neuromuscular function of the hindlimbs and increased the number of surviving MNs in the treated female SOD1^G93A^ mice. SA4503 tended to improve motor function and preserved neuromuscular junctions (NMJ), but did not improve MN survival. Western blot analyses revealed that the autophagic flux and the endoplasmic reticulum stress, two pathways implicated in the physiopathology of ALS, were not modified with Sig-1R treatments in SOD1^G93A^ mice. In conclusion, Sig-1R ligands are promising tools for ALS treatment, although more research is needed to ascertain their mechanisms of action.

## Introduction

Amyotrophic lateral sclerosis (ALS) is a fatal neurodegenerative disorder characterized by the progressive loss of upper and lower motoneurons (MNs), causing muscle paralysis and early death. Despite persistent efforts to develop treatments for this disease, no effective cure is available for ALS patients. Riluzole and edaravone are the only drugs approved by the FDA, but they have limited therapeutic benefits increasing the lifespan a few months (Edaravone (MCI-186) ALS 19 Study Group, 2017; Ludolph and Jesse, 2009). The exact pathophysiological mechanisms contributing to MN degeneration in ALS remain unclear. Nevertheless, the generation of transgenic animal models carrying ALS-related mutations has accelerated the research on physiopathology and preclinical therapeutic assays for ALS. Since the first mutation identified in familiar ALS was in the SOD1 gene (Rosen et al., 1993), the SOD1^G93A^ mouse is the most widely used ALS model, which develops the main clinical, electrophysiological and histopathological features of both familial and sporadic forms of the disease (Turner and Talbot, 2008; Mancuso et al., 2011b). Despite mutations in SOD1 gene are present in 20% of familiar ALS cases and only 2% of all ALS cases (Ragagnin et al., 2019), the interest of the SOD1 transgenic models is increased because alterations in SOD1 protein also occur in sporadic ALS cases (Bosco and Landers, 2010), and accumulation of wild-type SOD1 causes ALS (Graffmo et al., 2013).

Sigma-1 receptor (Sig-1R) is a protein ubiquitously expressed in the central nervous system (CNS) (Langa et al., 2003) and particularly enriched in the MNs. It is located in the endoplasmic reticulum (ER) cisternae at postsynaptic sites of C-terminals and at the mitochondria associated-endoplasmic reticulum membrane (MAM), an active and dynamic site in which there is crosstalk between mitochondria and ER (Hayashi and Su, 2007; Mavlyutov et al., 2012). Sig-1R is involved in numerous cellular processes, such as ion channel modulation, protein and lipid transport, ER stress response and mitochondria function (Penke et al., 2018; Herrando-Grabulosa et al., 2021). In recent years, genetic analyses revealed Sig-1R gene mutations involved in a juvenile form of ALS (Al-Saif et al., 2011; Watanabe et al., 2016) and in forms of motor neuropathies (Almendra et al., 2018; Ververis et al., 2019). Moreover, either accumulation of mutant SOD1 or absence of Sig-1R induced MAM disruption and mitochondrial dysfunction (Bernard-Marissal et al., 2015; Watanabe et al., 2016).

Nowadays, several Sig-1R ligands have interest as potential therapeutic agents against CNS disorders, including chronic neurological conditions such as pain, stroke, Huntington disease, among others (Bruna et al., 2018; Reilmann et al., 2019; Urfer et al., 2014). Regarding motoneuron diseases (MND), the Sig-1R agonist PRE-084 has shown positive effects reducing the MN death *in vitro* in organotypic culture of spinal cord subjected to excitotoxic damage (Guzmán-Lenis et al., 2009) and *in vivo* in the SOD1^G93A^ murine model of ALS (Mancuso et al., 2012), in the wobbler mouse model of spontaneous MN degeneration (Peviani et al., 2014), and after spinal nerve injury in adult mice and rats (Gaja-Capdevila et al., 2021; Penas et al., 2011). Studies testing two other Sig-1R agonists, SA4503 and pridopidine, also showed that treatment ameliorates ALS pathology (Ionescu et al., 2019; Ono et al., 2014), and pridopidine is being tested in a clinical trial for ALS (ClinicalTrials.gov NCT04615923). However, Sig-1R ligands may act differently, and even contrarily, on neuroprotective mechanisms by modulating calcium homeostasis (Tadić et al., 2017). Thus, the Sig1-R appears as a promising target to promote MN protection, but more studies are needed to establish the role of Sig-1R ligands in MND models, and the type of ligand that may be most effective. Considering the recent data, the aim of the work reported here was to comparatively evaluate the therapeutic efficacy of three Sig-1R ligands in an *in vitro* model of MN degeneration and in the SOD1^G93A^ mouse. Moreover, it was investigated whether the administration of Sig-1R ligands could promote the modulation of glial reactivity. Finally, regarding the importance of ER stress and autophagy in the ALS pathogenesis (Hetz and Saxena, 2017; Medinas et al., 2017; Nguyen et al., 2019), we investigated whether these Sig-1R ligands modulate these molecular pathways.

## Material and Methods

### Spinal Cord Organotypic Cultures

Spinal cord organotypic cultures (SCOCs) were prepared from lumbar sections of Sprague–Dawley pups (8–9 days-old) as previously described (Mòdol-Caballero et al., 2017). After harvesting, the spinal cord was cut in 350 μm thick transverse sections, that were transferred on Millicell-CM nets (0.4 μm, PICM03050, Millipore) and then into a six-well plate with the incubation medium [50% (v/v) minimal essential medium (MEM, M5775, Sigma), 2 mM glutamine, 25 mM HEPES, 25% (v/v) Hank’s Balanced Salt Solution (HBSS−/−, 14,175, Gibco) supplemented with 25.6 mg/ml glucose and 25% (v/v) heat-inactivated horse serum (26,050–088, Gibco), pH = 7.2). After 7 days *in vitro* (DIV), chronic excitotoxicity was induced by adding DL-threo-β-hydroxyaspartic acid (THA; 100 μM), a selective inhibitor of glutamate transport (Rothstein et al., 1993). The Sig-1R ligands were simultaneously co-added in the culture medium and renewed at each medium change twice per week. Sig-1R ligands PRE-084, BD1063 and SA4503 (Tocris) were tested at three different concentrations (30, 3 and 0.3 μM). Riluzole (5 μM) was also assayed as positive control. Slices were maintained for 28 DIV and then fixed with 4% paraformaldehyde in phosphate-buffered saline (PBS). The *in vitro* experiments have been performed in three independent cultures and resulting in 12 slices for each experimental condition.

### Animals and Experimental Design

Transgenic SOD1^G93A^ mice (B6SJL-Tg [SOD1-G93A] 1Gur) and non-transgenic wild type (WT) littermates were used. The transgenic offspring was identified by polymerase chain reaction (PCR) amplification of DNA extracted from the tail. Primer sequences were the following: hSOD1-forward CAT​CAG​CCC​TAA​TCC​ATC​TGA, hSOD1 reverse CGC​GAC​TAA​CAA​TCA​AAG​TGA, mIL2 forward CTA​GGC​CAC​AGA​ATT​GAA​AGA​TCT and mIL2 reverse GTA​GGT​GGA​AAT​TCT​AGC​ATC​ATC​C. Mice were maintained under standard conditions with access to food and water ad libitum. The experimental procedures were approved by the Ethics Committee of the Universitat Autònoma de Barcelona and followed the European Communities Council Directive 2010/63/EU.

The study included B6SJL female WT and SOD1^G93A^ mice divided in different experimental groups, either receiving vehicle or a Sig-1R ligand. We first performed a complete study in female mice, and after analyses, the study was also performed in male mice with two compounds, considering the differences in disease progression between sexes in this transgenic mouse. For the functional studies the following experimental groups of SOD1^G93A^ female mice were used: SOD1 + saline (n = 15), SOD1 + PRE-084 0.25 mg/kg (n = 14), SOD1 + BD1063 5 mg/kg (n = 12), SOD1 + SA4503 0.25 mg/kg (n = 7), SOD1 + SA4503 1 mg/kg (n = 7), in addition to untreated WT littermates (n = 15). Samples from these animals (n = 7–10) were collected for histological analysis at 16 weeks. Subgroups (n = 4–5) of WT, vehicle, PRE-084 and BD1063 groups were used for Western blot (WB) analyses at 8 and 16 weeks of age. For the functional studies in male the following groups of SOD1^G93A^ mice were used: SOD1 + saline (n = 10), SOD1 + PRE-084 (n = 10), SOD1 + BD1063 (n = 5), and untreated WT littermates (n = 10).

### Pharmacological Treatment

The Sig-1R ligands were given once a day from 8 to 16 weeks of age by intraperitoneal (i.p) administration of agonists PRE-084 (0.25 mg/kg, TOCRIS) and SA4503 (0.25 mg/kg and 1 mg/kg, TOCRIS) and the antagonist BD1063 (5 mg/kg, TOCRIS). The compounds were dissolved in saline solution, that was administered in the same volume to the untreated control group. The Sig-1R ligands were administered in a volume of 10 ml/kg.

### Electrophysiological Tests

Motor nerve conduction tests were performed at 8 weeks of age to obtain baseline values (prior to drug administration) to distribute them between the experimental groups, and then at 11, 13 and 16 weeks of age. Briefly, the sciatic nerve was stimulated by single pulses of 20 µs duration delivered at the sciatic notch. The compound muscle action potential (CMAP) was recorded from tibialis anterior (TA), gastrocnemius (GM) and plantar interossei (PL) muscles with microneedle electrodes (Mancuso et al., 2011b). The recorded potentials were amplified and displayed on a digital oscilloscope to measure the latency to the onset and the amplitude of the CMAP. Pentobarbital (50 mg/kg i. p.) was used to anesthetise the mice during the tests and mice body temperature was maintained by means of a thermostated heating pad.

### Locomotion Tests

The rotarod test was performed to evaluate motor coordination and balance of the animals, weekly from 8 to 16 weeks of age in SOD1^G93A^ and WT mice. Mice were placed onto the rod turning at 14 rpm, each mouse was given five trials and the longest latency without falling was recorded, with an arbitrary cut-off time of 180 s. The symptomatic disease onset for each mouse was determined as the first week when the mouse did not sustain 180 s on the rod.

### Histological and Immunohistochemical Analyses

SCOC were fixed, blocked with 5% normal horse serum in 0.3% Triton X-100 PBS solution (PBS-Tx), and incubated with primary antibody mouse anti-neurofilament H non-phosphorylated (SMI-32, 1:500; 801701, BioLegend) for 48 h at 4°C. Then, after several washes with 0.1% Tween-20 in PBS (PBS-Tw), slices were incubated with secondary antibody Alexa Fluor 488 donkey anti-mouse (1:500; A-2102, Invitrogen) for 2 h at RT. Cell nuclei were labeled with DAPI (1:5000; D9563, Sigma) and the sections were mounted with Fluoromount-G medium (SouthernBiotech). Images of the ventral horns were captured with a confocal microscope (LSM 700 Axio Observer, Carl Zeiss 20x/z0.5). The Cell Counter plugin of ImageJ software was used for quantifying SMI-32 positive neurons (MN survival) in each spinal cord.

At 16 weeks of age mice were sacrificed with an overdose of pentobarbital sodium and transcardially perfused with 4% paraformaldehyde in PBS. The lumbar segment of spinal cord was post-fixed for 2 h and cryopreserved in 30% sucrose solution in PB, whereas the hindlimb muscles were directly cryopreserved. For assessing MN survival, the spinal cord was serially cut in 20 µm thick transverse sections using a cryostat (Leica) and collected sequentially on series of 10 slides. Slices corresponding to L4-L5 spinal cord sections separated 100 µm were stained for 3 h with an acidified solution of 3.1 mM cresyl violet. Then, the slides were washed, dehydrated and mounted with DPX. MNs were identified by localization in the lateral ventral horn and strict morphological and size criteria: polygonal shape, prominent nucleoli and diameter larger than 20 µm.

For immunofluorescence analysis, lumbar spinal cord sections were blocked with blocking solution (10% normal donkey serum (NDS) and 0.2 mM glycine in PBS-Tx) for 1 h at RT, and then incubated overnight at 4°C with primary antibodies: anti-Iba1 (1:500; 019–19,741, Wako), anti-GFAP (1:500; 13–0300, Invitrogen), SQSTM1/p62 (1:150; 155686, Abcam). After several washes, sections were incubated for 2 h at RT with the corresponding secondary antibody: Alexa Fluor 488-conjugated secondary antibody (1:500; A-21206, Invitrogen), Cy3-conjugated secondary antibody (1:500; AP182C, Millipore), Alexa Fluor 594-conjugated secondary antibody (1:300; A-21207,Invitrogen) or Alexa Fluor 647-conjugated secondary antibody (1:300; Ab150155,Abcam). NeuroTraceTM 500/525 Green Fluorescent Nissl (1:200; N21480, Invitrogen) and DAPI (1:2000; D9563, Sigma) were used to stain MNs and nuclei, respectively. Finally, samples were washed in PB and mounted with Fluoromount-G. To quantify the glial cell reactivity images of the ventral horn were captured at x40 under the same conditions (sensitivity and exposure time) for each analyzed marker, using fluorescence microscopy (Olympus BX51, Japan). Fluorescence signal intensity (Integrated density) was analyzed using ImageJ software after defining a threshold for background correction. To quantify p62/SQSTM1 immunolabeling, photographs of the ventral horn were taken with confocal microscopy (LSM 700 Axio Observer, Carl Zeiss, 40xOil/z0.5). Integrated density of p62/SQSTM1 was quantified in a total of more than 50 MNs for each animal using a ROI manage tool from ImageJ software. The p62/SQSTM1 integrated density was analyzed in the glia by quantifying the whole slide image and subtracting the intensity of the MNs.

For neuromuscular junctions (NMJ) labeling, GM muscle was serially cut in 50 µm thick longitudinal sections and collected in sequential series. After blocking, the sections were incubated for 48 h at 4°C with the primary antibodies anti-neurofilament 200 (NF200, 1:1000; AB5539, Millipore, United States) and anti-synaptophysin (1:500; AB32127, Abcam). After washes sections were incubated overnight with Alexa Fluor 594-conjugated secondary antibody (1:200; A11042-A21207, Invitrogen) and Alexa Fluor 488-conjugated alfa-bungarotoxin (1:500; B13422, Life Technologies). Slides were mounted in Fluoromount-G with DAPI. Images were captured by confocal microscopy (LSM 700 Axio Observer, Carl Zeiss, 40xOil/z0.5). The proportion of innervated NMJs was calculated by classifying each endplate as occupied or vacant. Four fields with a total of more than 60 endplates were analyzed per each mouse.

### Protein Extraction and Western Blot Analysis

At 8 or 16 weeks of age, mice were euthanized with an overdose of pentobarbital sodium. The lumbar spinal cord from WT and SOD1^G93A^ of each experimental group was harvested and frozen in liquid nitrogen for storage. To lysate samples the RIPA lysis buffer with protease inhibitor cocktail (Sigma) and phosphatase inhibitors (PhosphoSTOP Roche) was used. Then, samples were sonicated and centrifuged at 12,000 rpm for 10 min at 4°C. Finally, total protein concentration was determined by the BCA Protein Assay Kit (Biorad). 20–30 μg of protein per sample were loaded into 7.5–15% SDS-polyacrylamide gels and transferred into a PVDF membrane. After blocking, primary antibodies were incubated at 4°C overnight (Table 1). After incubation with appropriate Horseradish peroxidase (HRP)-coupled secondary antibody (1:5000; Bio-rad) for 1 h at RT, proteins were visualized using the Clarity Western ECL Substrate (Cat#1705061, Bio-Rad Laboratories). Images were collected using a transilluminator (Chemidoc MP Imaging System, BioRad) and blots were analyzed using the Lane and band plugin of Image Lab software (Bio Rad). Data were normalized first by the loading control (actin or tubulin) and afterwards by the mean of the control (WT) samples (n = three to six samples were analyzed per each condition and time-point of the study).

**TABLE 1:** Primary antibodies used for WB experiments.

Antibody name	Dilution	Description	References number
LC3B	1:500	Rabbit polyclonal	#ab51520; Abcam
Beclin 1	1:1000	Rabbit polyclonal	#3738; CST
XBP-1	1:300	Rabbit polyclonal	#37152; Abcam
GADD153/CHOP	1:500	Mouse monoclonal	#sc-7351; SCBT
Sigma-1 Receptor	1:250	Rabbit polyclonal	# 223,702; Abcam
GRP78/BiP	1:500	Rabbit polyclonal	#G8918; Sigma-Aldrich
IRE1 (phospho S724)	1:250	Rabbit polyclonal	# ab48187; Abcam
IRE1α	1:500	Rabbit polyclonal	#3294; CST
β-Actin	1:10,000	Mouse monoclonal	#A5316; Sigma-Aldrich
α-tubulin	1:10,000	Mouse monoclonal	#T9026; Sigma-Aldrich

### Statistical Analysis

GraphPad Prism 8 software was used to perform data analyses, all data is expressed as mean ± SEM. Electrophysiological and functional measurements were analyzed with repeated measurements Two-Way ANOVA and histological and molecular data were analyzed using One-way ANOVA. Bonferroni test was used as the post hoc test for multiple comparisons. Differences were considered significant at *p* ≤ 0.05.

## Results

### Sig-1R Ligands Exert Neuroprotection in SCOCs Under Chronic Excitotoxicity

**FIGURE 1:**
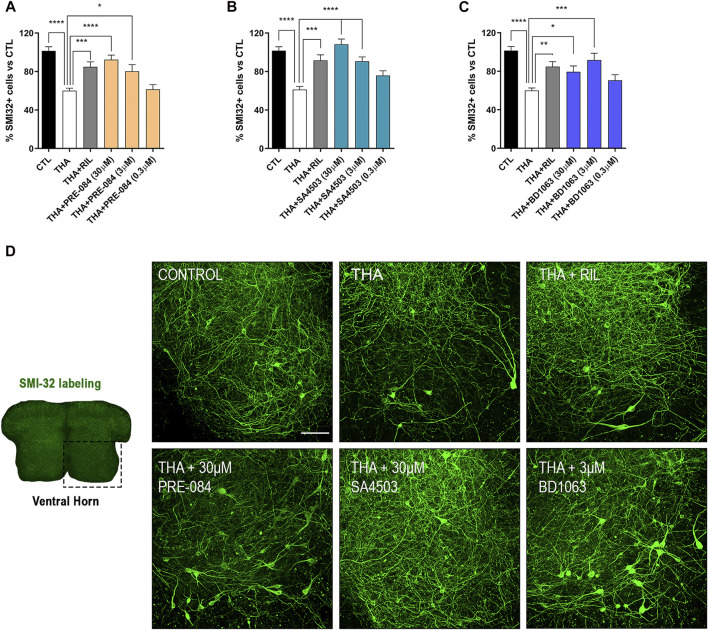
PRE-084, BD1063 and SA4503 prevent MN death under chronic excitotoxicity *in vitro* **(A-C)** Plots showing the percentage of SMI-32 positive cells in the ventral horn of spinal cord hemislices under excitotoxicity by THA and the addition of Sig-1R ligands (mean ± SEM; n = 16–24 hemisections per treatment). One-way ANOVA followed with Bonferroni’s post hoc test: ****p < 0.0001, ***p < 0.001; **p < 0.01, *p < 0.05 vs THA alone condition **(D)** Microphotograph of a SCOC slice labeled with SMI-32 antibody, and representative images of the ventral horn at 28 DIV of all tested conditions. Scale bar 100 μm.

Addition of THA to the SCOC induced a significant reduction of about 40% in the number of SMI-32 labeled MNs in the ventral horn after 21 DIV, compared to control slices (Figure 1). Slices treated with THA and PRE-084, BD1063 or SA4503 showed significant preservation of MNs at the two doses tested. The Sig-1R agonists PRE-084 and SA4503 significantly reduced MN death in a dose-dependent manner, with maximal protective effect at 30 μM (Figures 1A,B). The antagonist BD1063 also prevented MN death, with a highest effect at 3 μM (Figure 1C). Furthermore, the positive control against excitotoxicity Riluzole presented significant MN protection at similar levels than the Sig-1R ligands tested.

### Treatment With Sig-1R Ligands Preserves Neuromuscular Function in SOD1^G93A^ Mice

**FIGURE 2:**
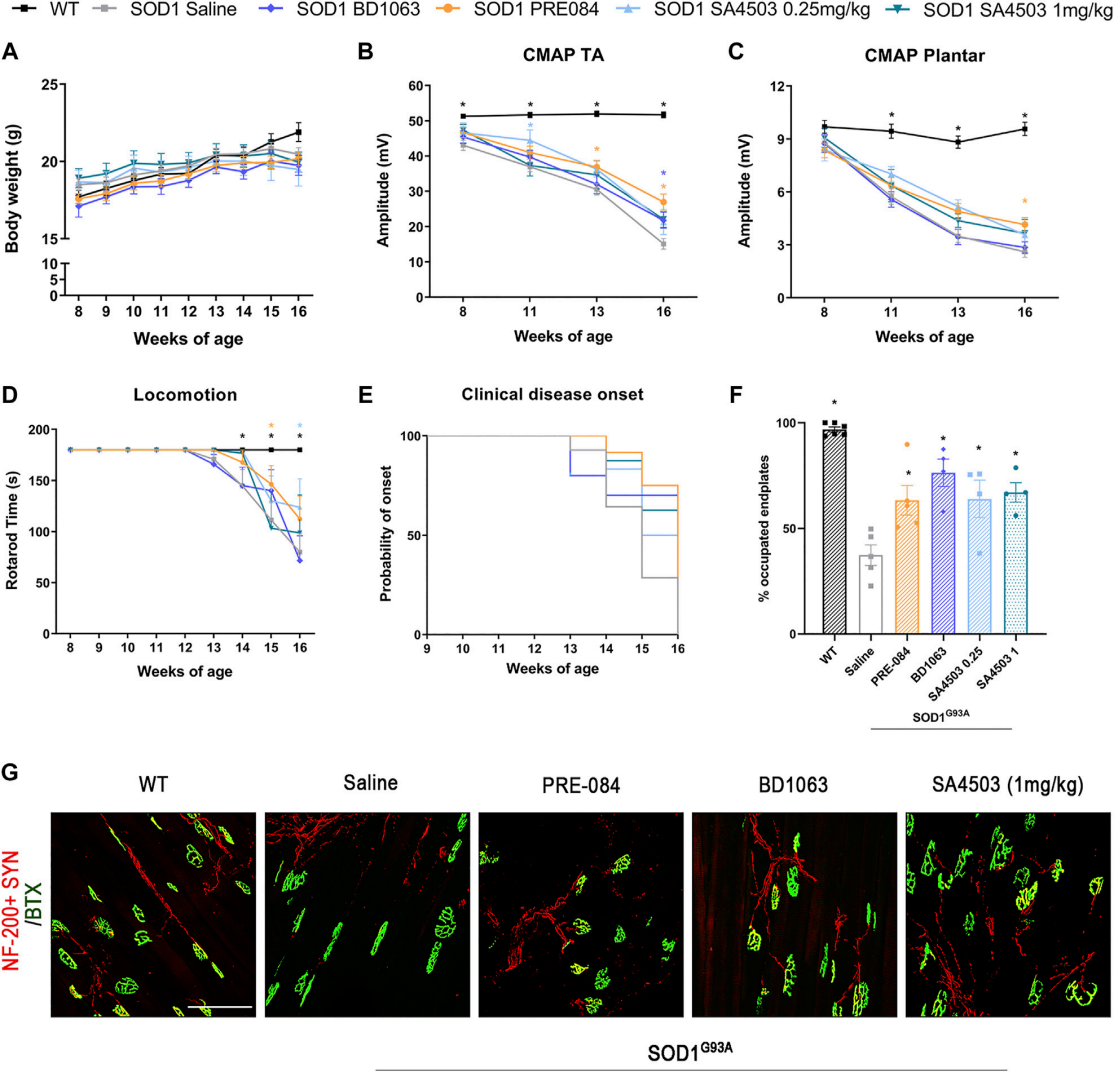
Treatment with PRE-084, BD1063 and SA4503 enhances neuromuscular function in SOD1^G93A^ female mice **(A)** Body weight of mice was monitored weekly during the 8 weeks of the follow-up **(B-C)** Values of the amplitude of compound muscle action potentials (CMAP) of tibialis anterior (TA) and plantar interosseus muscles **(D)** Graph showing the effect of the different Sig-1R treatment on functional outcome assessed with the rotarod test (n = 15 WT; n = 15 SOD1 saline; n = 12 SOD1 BD1063; n = 7 SOD1 SA4503 0.25 mg/kg; n = 7 SOD1 SA4503 1 mg/kg, and n = 14 SOD1 PRE-084 mice) **(E)** Probability of clinical onset of disease evaluated by the fall in the rotarod test. Some Sig-1R ligands delayed the onset of locomotion deficits but without significant differences **(F)** Plot of the percentage of innervated NMJ (overlap of signals) in the different experimental groups (n = four to six mice per group) **(G)** Representative confocal images of GA NMJs at 16 weeks of age. The maximum projection images shown were generated from 1.3 µm z projections. Scale bar 100 μm. Data are mean ± SEM, analyzed by One-way **(F)** and Two-way **(A-D)** ANOVA with Bonferroni’s multiple comparisons test. *p < 0.05 vs SOD1^G93A^ saline mice.

SOD1^G93A^ mice treated with Sig-1R ligands for 8 weeks maintained a gain of body weight throughout the study, and did not present any secondary effect, indicating lack of general toxicity of these ligands (Figure 2A).

In order to assess the effect of Sig-1R ligands on neuromuscular function of the SOD1^G93A^ mice, we performed motor nerve conduction tests during the follow-up. Results showed that SOD1^G93A^ mice treated with PRE-084 and with BD1063 had a significantly higher TA and GM CMAP amplitude compared to saline administered SOD1^G93A^ mice at 13–16 weeks of age (Figure 2B and Supplementary Figure S1A). CMAP amplitude differences did not reach statistical significance in SA4503 treated group compared with untreated mice at the end of the follow up. Male SOD1^G93A^ treated with PRE-084 had a significant preservation of TA and GM CMAP amplitude throughout the follow-up compared to the saline group, while BD1063 treatment has lower effect (Supplementary Figure S1B, C). The PL CMAP amplitude showed mild preservation for groups treated with PRE-084 and SA4503 at both doses, although it was only significant for PRE-084 (Figure 2C).

The rotarod test revealed that SOD1^G93A^ mice treated with SA4503 at dose of 0.25 mg/kg and PRE-084 significantly improved the functional outcome compared with the untreated group, but animals treated with BD1063 and SA4503 1 mg/kg did not have any improvement (Figure 2D). Furthermore, the disease onset was delayed by 1 week (14 weeks of age) in mice treated with SA4503 (0.25 and 1 mg/kg) and PRE-084 versus the saline group (13 weeks) (Figure 2E).

The innervation of NMJ of the GM muscle was evaluated at 16 weeks of age. All the SOD1^G93A^ mice treated with Sig-1R ligands had a significantly higher number of innervated endplates compared with the saline group, supporting the preservation of CMAP amplitude observed in the nerve conduction tests (Figures 2E,F).

### Sig-1R Ligands Promote MN Survival and Reduce Microglial Reactivity in SOD1^G93A^ Mice

**FIGURE 3:**
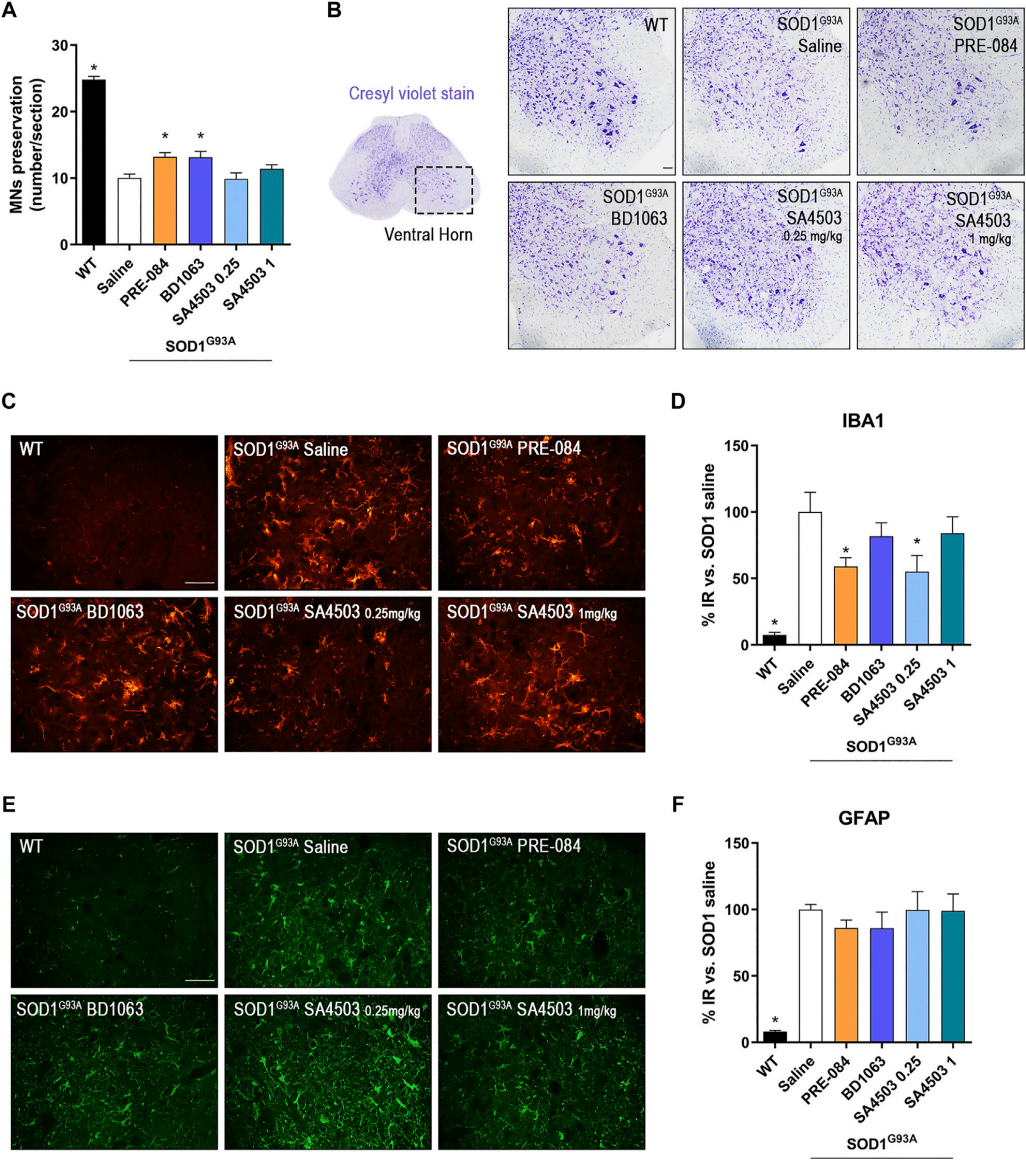
PRE-084 and BD1063 reduce spinal MN degeneration and decrease microgliosis in SOD1^G93A^ female mice at 16 weeks of age **(A)** Quantification of surviving MNs (mean number of MNs per section ±SEM) in the lumbar spinal cord, showing improved MN preservation with Sig-1R ligands PRE-084 and BD1063. **(B)** Representative spinal cord images of MNs stained with cresyl violet. Scale bar 100 μm. **(C, E)** Representative images of immunoreactivity for Iba1 **(C)**, a marker for microglia, and GFAP **(E)**, a marker of astrocytes, in the ventral horn of WT and SOD1^G93A^ mice with or without Sig-1R treatments. Scale bar 50 μm. **D, F)** Graphs showing the quantification of percentage of Iba-1 **(D)** and GFAP **(F)** immunolabeling in ventral horn of spinal cord (n = 10 WT, n = 10 SOD1 saline, n = 8 SOD1 BD1063, n = 7 SOD1 SA4503 0.25 mg/kg, n = 7 SOD1 SA4503 1 mg/kg and n = 10 SOD1 PRE-084 mice). *p < 0.05 vs SOD1^G93A^ saline mice.

The quantification of α-MNs in the ventral horn of lumbar spinal cord sections stained with cresyl violet at 16 weeks of age, revealed that treatment with PRE-084 and BD1063 mildly but significantly prevented the death of spinal MNs at the end-stage of the disease in comparison with untreated SOD1^G93A^ mice, whereas SA4503 at both doses assessed did not have a noticeable effect (Figures 3A,B).

Microglial and astroglial response was found markedly increased in SOD1^G93A^ compared to WT mice. Treatment with Sig-1R ligands reduced microglial reactivity, although only PRE-084 and SA4503 at 0.25 mg/kg caused a significant decrease, whereas the other treatments did not reach statistical significance (Figures 3C,D). Regarding astroglial immunoreactivity in the spinal cord ventral horn, we found that it was not modified by administration of the Sig-1R ligands in female SOD1^G93A^ mice (Figures 3E,F).

### Analyses of Autophagic Flux and ER Stress During the Progression of ALS With Sig-1R Ligands

**FIGURE 4:**
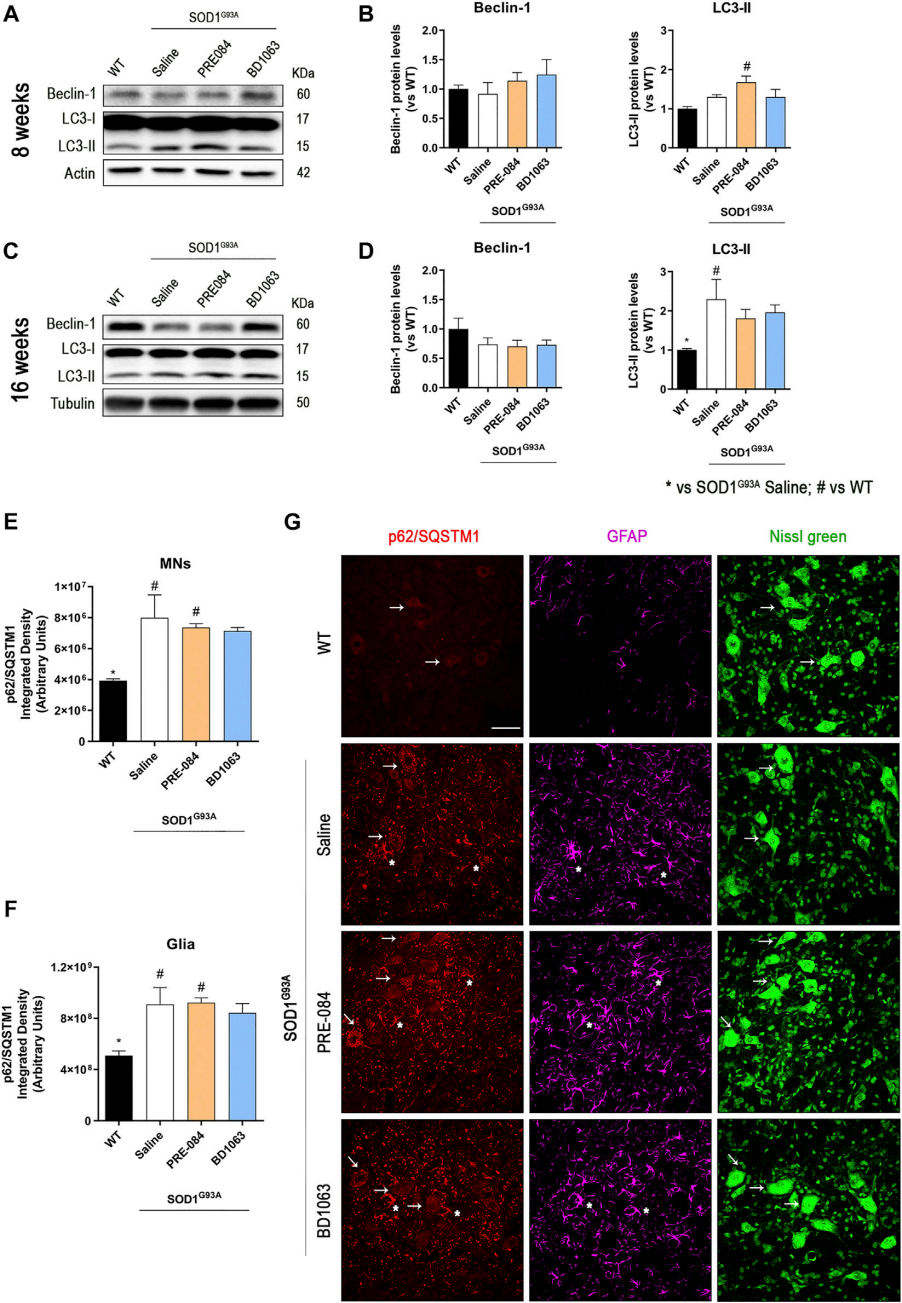
Protein levels of autophagic flux markers in SOD1^G93A^ female mice. Representative blots and protein level quantification of the autophagic markers Beclin-1 and LC3-II at 8 **(A, B)** and 16 **(C, D)** weeks of age. Quantification of the p62/SQSTM1 immunolabeling in the MNs **(E)** and in the glia (whole image without MNs) **(F)**. **(G)** Representative images of p62/SQSTM1 (red), GFAP (cyan) and FluoroNissl (green) immunofluorescence in the ventral horn of lumbar spinal cord of WT and SOD1^G93A^ mice at 16 weeks. White arrows show examples of MNs with cytosolic p62 accumulation and asteriscs show p62 immunolabeling in the astroglia. Scale bar 50 μm. Data is mean ± SEM; n = three to five mice per group. One-way ANOVA followed with Bonferroni’s post hoc test for multiple comparison. *p < 0.05 vs SOD1^G93A^ saline, #*p* < 0.05 vs WT.

Considering that PRE-084 and BD1603 significantly preserved spinal MNs, we performed WB analyses of lumbar spinal cord lysates from WT and SOD1^G93A^ saline, PRE-084 and BD1063 treated mice at 8 and 16 weeks of age to evaluate two main molecular pathways implicated in ALS pathogenesis. Three markers of autophagy flux (Figure 4) were analyzed and we found that there was no differences in Beclin-1 protein levels between WT and SOD1^G93A^ mice groups at the two time points evaluated. A progressive increase of LC3-II levels was observed in SOD1^G93A^ saline group, observing higher levels at 16 weeks of age compared with WT mice group. In contrast, at 8 weeks of age, higher LC3-II levels were detected in PRE-084 group that were maintained at 16 weeks. No differences regarding LC3-II protein levels were detected in BD1063 group (Figures 4A–D). Immunofluorescence analyses of lumbar spinal cord sections revealed a significant accumulation of p62/SQSTM1 immunoreactive dots in the MNs and glial cells, mainly astroglia, of SOD1^G93A^ animals at 16 weeks of age (Figure 4E–G). In summary, treatment with Sig-1R ligands PRE-084 and BD1063 did not markedly modify the protein levels of autophagic markers in comparison with saline SOD1^G93A^ mice.

**FIGURE 5:**
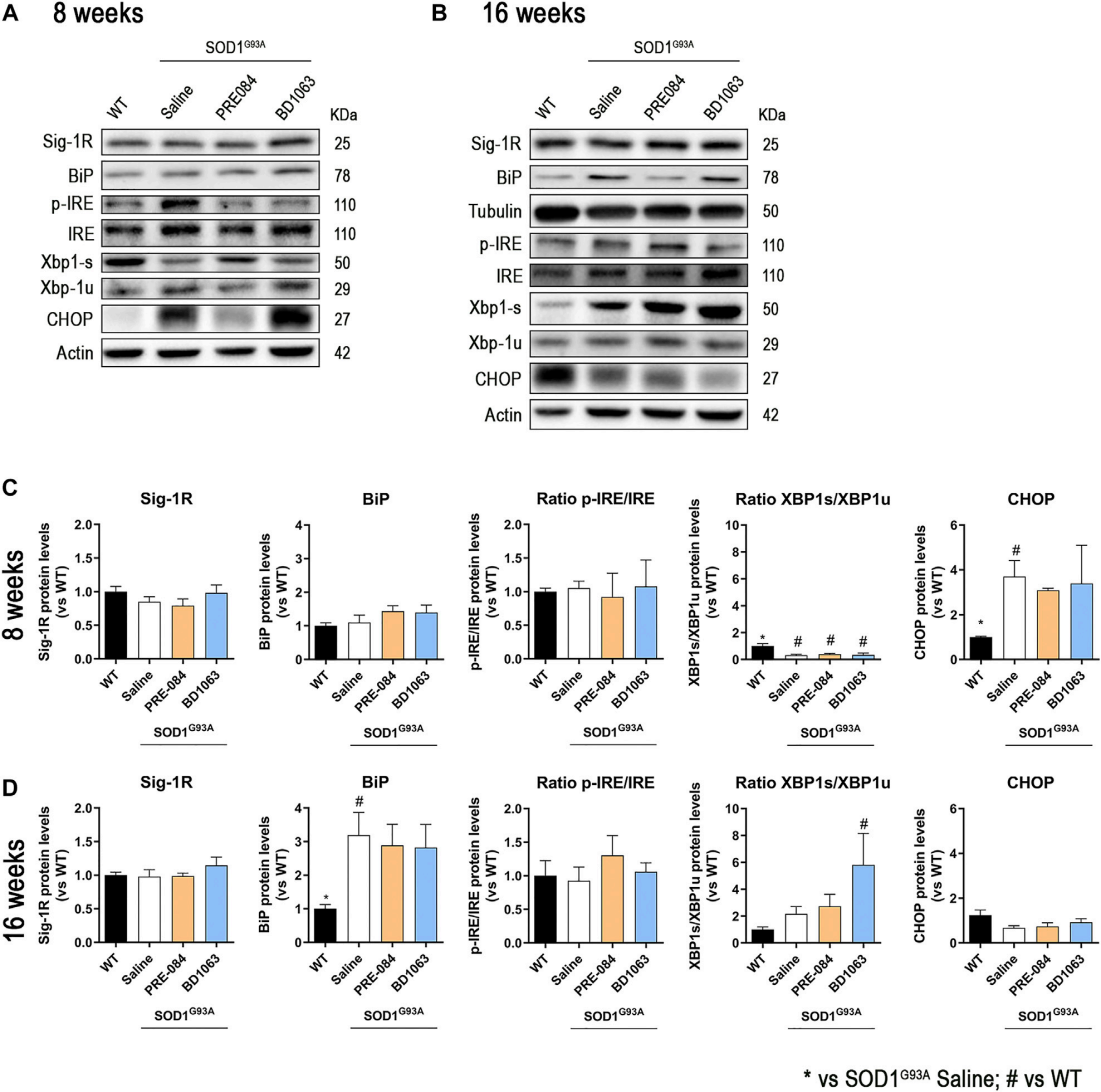
Protein levels of ER stress markers in SOD1^G93A^ female mice. Representative blots of ER stress markers at 8 **(A)** and 16 **(B)** weeks of age. Quantification of protein levels of Sig-1R, BiP, ratio p-IRE1/IRE1, ratio XBP1s/XBP1unspliced and CHOP at 8 **(C)** and 16 **(D)** weeks of age. Data is mean ± SEM; n = three to five mice per group. One-way ANOVA followed with Bonferroni’s post hoc test for multiple comparison. *p < 0.05 vs SOD1^G93A^ saline, #*p* < 0.05 vs WT.

We also monitored the levels of ER stress markers in the spinal cord (Figures 5A,B). The protein levels of Sig-1R did not change at 8 and 16 weeks in SOD1^G93A^ mice, as previously described (Mancuso et al., 2012), and administration of Sig-1R ligands PRE-084 and BD1063 did not modify the levels of this receptor (Figures 5C,D). During disease progression, WB analyses revealed a marked increase in the chaperone BiP at 16 weeks of age in SOD^G93A^ mice (Figure 5D). There were no differences in the ratio p-IRE1/IRE between experimental groups at both time points evaluated, whereas there was a significant reduction of the ratio XBP1s/XBP1unspliced at 8 weeks, that was reverted to increased levels at 16 weeks in SOD1^G93A^ mice, with a significant difference for the BD1063 treated compared to WT mice. We also observed a significant increase of CHOP levels in SOD1^G93A^ mice at 8 weeks of age, though no differences were found at 16 weeks of age.

## DISCUSSION

The results of this study demonstrate the beneficial effects of the Sig-1R agonist PRE-084 as well as the Sig-1R antagonist BD1063, on preserving neuromuscular function and attenuating MNs loss in the SOD1^G93A^ mouse model of ALS. The compound SA4503 showed lower effects in ameliorating the progression of the disease. Whereas previous studies have reported that PRE-084 (Mancuso et al., 2012) and SA4503 (Ono et al., 2014) treatment ameliorated the progression and survival of spinal MNs, we report for the first time that a Sig-1R antagonist, such as BD1063, has also neuroprotective effects in preclinical models of ALS. We have recently reported that all PRE-084, SA4503 and BD1063 also prevent MN death in a model of spinal root injury in mice (Gaja-Capdevila et al., 2021), emphasizing the interesting effects of Sig-1R ligands for improving motor neurodegenerative conditions.

Although similar outcomes were obtained in terms of MN preservation *in vitro* in the SCOC with the three Sig-1R ligands tested, there were noticeable differences in the *in vivo* model of ALS. Glutamate excitotoxicity is one of the main pathophysiological mechanisms implicated in ALS. Astrocytes are the main regulators of extracellular glutamate levels and excitatory amino acid transporter 2 (EAAT2) is the main astroglial synaptic glutamate reuptake transporter protein. The loss of EAAT2 has been reported in both ALS patients (Bristol and Rothstein, 1996) and rodent models (Howland et al., 2002). Our *in vitro* results showed that two considered Sig-1R agonists, PRE-084 and SA4503, and the antagonist BD1063 induced protection against chronic excitotoxicity, at the doses of 3–30 μM. Indeed, some reports reveal that binding to Sig-1R prevented neuronal death in *vitro* studies. Our group described that PRE-084 (at 10 μM) protected the SCOC against acute glutamate toxicity (Guzmán-Lenis et al., 2009). Ono and collaborators found that SA4503 (at 10 μM) protected the NSC34 cell line against SOD1^G93A^ and serum free neurotoxicity (Ono et al., 2014). However, those protective effects were inhibited by the co-addition of a Sig-1R antagonist, BD1063 or BD1047, respectively. In our study we assessed each compound alone, without combining them, demonstrating that BD1063 had also similar neuroprotective effects.

Several reports have focused on Sig-1R ligands to modulate ALS progression. PRE-084 (0.25 mg/kg) daily administrated in SOD1^G93A^ from 8 to 16 weeks of age showed neuroprotection, improving MN function and survival and extending the lifespan of SOD1^G93A^ mice (Mancuso et al., 2012). SA4503 (1 mg/kg) treatment from 5 weeks of age extended survival time, but did not affect the onset time in the SOD1^G93A^ mice (Ono et al., 2014). However, we found a delay of 1 week of disease onset compared to untreated mice. In addition, in our study we evaluate the number of MN survival to compare with the other two Sig-1R ligands. Even though SA4503 did not significantly preserve spinal MNs as PRE-084 and BD1063 treatment did, all the pharmacological treatments significantly preserved NMJ innervation in the hindlimb muscles at 16 weeks of age. This is a remarkable result, since NMJ disruption is an early event in ALS pathogenesis (Fischer et al., 2004; Mancuso et al., 2011a). Since SA4503 and PRE-084 had been shown to prolong the lifespan of SOD1^G93A^ mice, a further study is needed to elucidate whether BD1063 has similar effect. Recently, pridopidine, a small molecule that modulates axonal transport deficit and causes a reduction in mutant SOD1 aggregates in the spinal cord of SOD1^G93A^ mice though Sig-1R, was reported to attenuate also NMJ disruption (Ionescu et al., 2019). Taken together, it seems that Sig-1R ligands improve several cellular and histological hallmark pathologies related to ALS.

Despite the three Sig-1R compounds assessed, PRE-084, SA4503 and BD1063, have demonstrated potential to bind the Sig-1R, the neuroprotective effects observed in the SOD1^G93A^ mouse model significantly differed between the three ligands. In the same line, Wang and others compared the Sig-1R ligands SA4503, PRE-084 and pentazocine (PTZ) in a model of severe retinopathy (Wang et al., 2020). *In vitro* results yielded similar outcomes, whereas neither PRE-084 or SA4503 afforded *in vivo* protection comparable to PTZ. A wide range of evidence is now available to support the role of Sig-1R in the treatment of several CNS disorders, such Parkinson disease or ischemia (Nguyen et al., 2017; Haga et al., 2019; Zhao et al., 2019). It is important to note that in this study Sig-1R ligands were classified as agonists or antagonist according to the BiP/Sig-1R association assay, in which the interaction between Sig-1R and the chaperone BiP is used to identify the functional nature (agonistic or antagonistic) of compounds. However, classification of Sig-1R ligands causes a lot of controversy because some studies revealed different cellular function between compounds considered as agonists of Sig-1R. Indeed, Sig-1R ligands of the same type may act differently in each pathology/degenerative disease, even in opposite way (Tadić et al., 2017). For example, it has been shown that the agonist SA4503 normalized cytosolic Ca^2+^ levels following activation by kainate and by bradykinin in embryonic MNs, whereas PRE-084 (also Sig-1R agonist) did not exert any significant effect (Tadić et al., 2017). Furthermore, we emphasize that the traditional concept of agonist or antagonist is controversial for Sig-1R ligands, that may act as modulators of this receptor promoting activity in different pathways with a delicate balance of effects (for review see Herrando-Grabulosa et al., 2021). Moreover, the total absence of Sig-1R in SOD1^G93A^ ALS mice model accelerates the ALS pathology (Mavlyutov et al., 2013). Therefore, considering the positive results obtained, BD1063 might act pharmacologically as a partial agonist in our animal model. Further comparative studies with other ligands classified as antagonists such as BD1047 and studies related with ligand classification *in vivo* are needed.

A body of evidence has demonstrated the contribution of neuroinflammation, the role of non-neuronal cells including microglia and astrocytes in ALS pathogenesis. Genetic deletion of mutant SOD1 selectively in microglia increased the lifespan of ALS mice, despite the mutant protein was expressed in MNs and all other cell types (Boillée et al., 2006). We observed a reduction in microglia activation following treatment with PRE-084 and with SA4503 at a dose of 0.25 mg/kg (see Figure 3D), but not with BD1063. The effect of PRE-084 on reducing the microglial reactivity and improving the MN environment was already found in the SOD1^G93A^ ALS model (Mancuso et al., 2012), as well as in a mouse model of spinal muscular atrophy (SMA^2B/-^), in which PRE-084 treatment mitigated reactive gliosis restoring the altered M1/M2 balance (Cerveró et al., 2018). On the other hand, we did not observe any effect with any of the three Sig-1R ligands assessed on reactive astrogliosis. Contrarily, after spinal root injury, where MN death also occurs, PRE-084 reduced astroglia immunoreactivity (Penas et al., 2011; Gaja-Capdevila et al., 2021). All these studies highlight the role of Sig-1R ligands modulating the neuroinflammatory response.

The etiology underlying the development of ALS remains poorly understood, but abnormal protein aggregation and altered proteostasis are common features of sporadic and familial ALS forms (Medinas et al., 2017). To elucidate the mechanisms through which BD1063 and PRE-084 promote MN preservation we analyzed autophagic flux and ER stress. Autophagy is an intracellular lysosome degradation system responsible for the clearance of cytoplasmic components and organelles. Enhancement of autophagy has been reported in ALS with alterations in several steps. Thus, the level of LC3-II, which is correlated with the extent of autophagosome formation, was found increased in SOD1^G93A^ transgenic mice at symptomatic stage (Morimoto et al., 2007; Tian et al., 2011), as we also found in this study. The marker of the late stage autophagosome, the autophagy adaptor p62, interacts with polyubiquitinated misfolded mutant SOD1 (mSOD1), sequestering mSOD1 into protein inclusions, so fusion of the autophagosome to the lysosome becomes insufficient at the end stage (Tian et al., 2011). Furthermore, autophagy is activated in the ventral spinal cord MNs in sporadic ALS patients, observed by immunostaining for LC3 and p62 (Mizuno et al., 2006; Sasaki, 2011). In our results, we also observed that there was an accumulation of SQSTM1/p62 in the MNs of spinal cord and in the glia cells in SOD1^G93A^ mice. However, there was no difference between untreated group and animals treated with the Sig-1R ligands. It is worth to mention that in ALS the role of autophagy is confusing and it is still unknown whether activation or inhibition of autophagy may influence in the disease progression (Nguyen et al., 2019), and depending on the model used (Medinas et al., 2017).

On the other hand, increased expression of ER stress markers was observed in post-mortem tissues from ALS patients (Hetz et al., 2009), and correspondingly, in this work we observed up-regulation of BiP and XBP1s in the spinal cord of symptomatic SOD1^G93A^ mice. The unfolded protein response (UPR) and the autophagy pathway have been linked (Hetz et al., 2009); thus, XBP1 conditional deletion in the nervous system ameliorated SOD1 mice pathogenesis, through up-regulation of the autophagy pathway boosting the degradation of mSOD1 aggregates. In the context of ALS, the functional significance of ER stress is still unclear, because activating UPR is a protective response to increase protein folding and quality control mechanisms, whereas chronic stress may represent a deleterious signaling due to irreversible disturbance of ER homeostasis. Although we found some differences in the markers analyzed by WB between WT and SOD1^G93A^ mice, we did not observe changes induced by treatment with the Sig-1R ligands, indicating that autophagy and ER stress pathways are not significantly modified through Sig-1R modulation. However, specific immunolabeling analyses of the spinal cord may reveal changes in specific populations, either MNs or glial cells, that may be obscured in protein analyses of the whole spinal cord tissue. Thus, further experiments are needed to elucidate the mechanisms of action by which Sig-1R ligands yield neuroprotective effects.

## Data Availability

The data that support the findings of this study are available from the corresponding author under reasonable request.
