# Supplementary material for: Sigma-1 Receptor is a Pharmacological Target to Promote Neuroprotection in the SOD1G93A ALS Mice
Source: Front Pharmacol. 2021 Dec 10;12:780588. doi: 10.3389/fphar.2021.780588 (PMC8702863; doi:10.3389/fphar.2021.780588)
Supplement: Supplementary file 1 [file DataSheet1.PDF]

Supplementary Material

Supplementary Figures

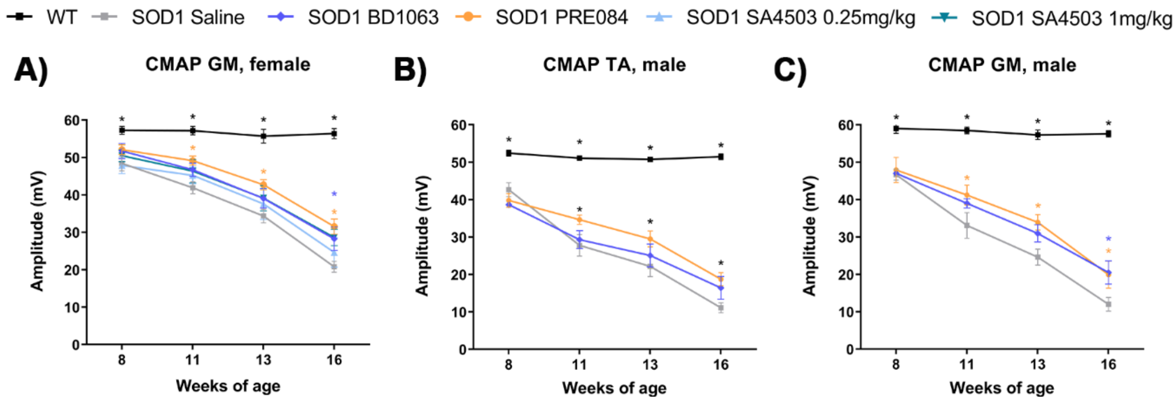

Supplementary Figure 1. Motor nerve conduction test of SOD1^{G93A} female and male mice treated with Sig-1R ligands. **(A)** Plot showing the amplitude of the compound muscle action potential (CMAP) in gastrocnemius (GM) muscle in females during the follow-up. **(B-C)** Plots showing the amplitude of CMAP in the tibialis anterior (TA) and GM muscle in males during the follow-up. (n=10 WT, n=10 SOD1 saline, n=5 SOD1 BD1063 and n=10 SOD1 PRE-084 mice). Data are mean ± SEM, analysed by Two- way ANOVA with Bonferroni's multiple comparisons test. *p< 0.05 vs. SOD1^{G93A} saline mice.
